# A biodegradable film based on cellulose and thiazolidine bearing UV shielding property

**DOI:** 10.1038/s41598-022-11457-5

**Published:** 2022-05-12

**Authors:** Rasha A. Baseer, Sawsan Dacrory, Mohamed A. M. El Gendy, Ewies F. Ewies, Samir Kamel

**Affiliations:** 1 Department of Polymers and Pigments technology, Chemical Industries Research Institute, National Research Centre, 33ElBohouth St., (Former El Tahrir), Dokki, 12622 Giza Egypt; 2 Cellulose and Paper Department, Chemical Industries Research Institute, National Research Centre, Cairo, 12622 Egypt; 3 Drug Bioassay-Cell Culture Laboratory, Pharmacognosy Department, Pharmaceutical and Drug Industries Research Institute, National Research Centre, 33 ElBohouth St., (Former El Tahrir), Dokki, P.O. 12622 Giza Egypt; 4 Organometallic and Organometalloid Chemistry Department, Chemical Industries Research Institute, National Research Centre, 33 ElBohouth St., (Former El Tahrir), Dokki, P.O. 12622 Giza Egypt

**Keywords:** Biomaterials, Materials for optics, Materials chemistry, Organic chemistry, Polymer chemistry

## Abstract

The current rationale is exploring new eco-friendly UV- shielding films based on cellulose and thiazolidine. Cellulose was oxidized to dialdehyde cellulose (DAC) and tricarboxy cellulose (TCC) by periodate and TEMPO/periodate/hypochlorite, respectively. While *E*-3-amino-5-(phenyldiazenyl)-2-thioxothiazolidin-4-one (TH) was synthesized by coupling diazonium salt with the 5-methylene of 2-thioxo-4-thiazolidinone. DAC was then coupled with TH via Schiff base reaction and incorporated onto TCC with different ratios to get UV-shielding films. ^1^HNMR, infrared spectroscopy (FTIR), and thermal gravimetric analysis (TGA) were used to investigate the chemical structure of the synthesized materials. In addition, the films' morphology, thermal, mechanical, and UV-shielding properties were investigated. The UV-shielding studies revealed that the film with 10% DAC-TH has 99.88, 99.99, and 96.19% UV-blocking (UVB), UV-absorbance (UVA), and Ultra-violet protection (UPF), respectively. Moreover, the prepared films demonstrated promising antimicrobial activity against *Escherichia coli, S. aureus, P. aeruginosa,* and *Candida albicans.* Finally, the prepared films showed no cytotoxic effects on normal human skin fibroblast's HFB-4 cell line.

## Introduction

For the last decades, polymer films have been widely applied as packaging materials in many primary industries such as food, drugs, and electronic devices for their significant low cost, lightweight, good elasticity, and high transmittance^1,2^. However, scientists have turned to create biodegradable materials with the mounting voices calling for environmental conservation and the trend to use friendly materials^3,4^. These materials, obtained from renewable sources, are pure polymer, composite or blended products, and they could be classified as green polymer. Recently, cellulosic materials have emerged as a strong alternative to synthetic polymers due to their unique biocompatible, biodegradable, non-toxic, and allergen-free^5^. Depending on its functional group that can be inserted into its chains backbone via the suitable modification. It looks most promising material and plays an important role in many applications such as drug delivery^6^, conductive materials^7,8^, Fertilizers agents^9^, and water treatment^10^. Dialdehyde cellulose (DAC) is the most widely utilized oxidized cellulose with highly reactive sites. Periodate is widespread to oxidize cellulose to dialdehyde cellulose^11,12^. It oxidizes the vicinal hydroxyl groups of cellulose at C2 and C3^6^. In recent years, dialdehyde cellulose has received increasing attention as an ideal crosslinking agent whose aldehyde groups can crosslink with NH_2_ of the amino acid via Schiff's base interaction^7^.

On the other hand, the building of UV-block cellulose films became an urgent need for further development and applications as biodegradable and eco-friendly UV-shielding materials^13,14^. Many approaches have been performed to develop UV-shielding cellulose through thermal treatment technology, presented as a green and straightforward approach for preparing UV-block cellulose. However, this method depends on the yellow discoloration of cellulosic fibers during the treatment^15,16^. Moreover, UV absorbers are considered the dominant technology to achieve high UV protection via incorporating them into cellulose films. In this context, the development of highly efficient cellulose-based UV-shielding films has the scientist's great attention^17^. Organic and inorganic UV-absorbers are mostly incorporated into cellulosic films to achieve UV protection depending on the type of functional group inserted in its chain backbone^13^.

On the other hand, heterocyclic compounds can be found in natural and synthetic materials and are mainly known for their significant UV-absorbing property^18–20^. 3-amino-2-thioxothiazolidin-4-one, commonly known as rhodanine, has a broad spectrum of biological activities demonstrating antiseptic, anti-inflammatory, antiparasitic, antifungal, antidiabetic, antiviral, and antineoplastic activity^21–23^. Many studies were performed on rhodanine derivatives to prove antimicrobial activity^24,25^. Also, their azo dyes derivatives are used as a spectrophotometric chemosensor for Fe^3+^^26^ with optical properties^27^.

Based on this survey herein, we aim to synthesize E-3-amino-5-(phenyldiazenyl)-2-thioxothiazolidin-4-one (TH) and couple it with dialdehyde cellulose (DAC) to exploit the dual π bond of each of the aryl azo group, thioxothiazolidin-4-one, and the aldehyde oxime of DAC resulted from Schiff base beside to the lone pair of nitrogen, sulfur, oxygen which overlap arising to the splitting of the originally degenerate n orbitals to promote high UV absorbance material. It is incorporated with different ratios into tricarboxylic cellulose (TCC) to formulate biodegradable and antibacterial film with UV-shielding and high safety when tested on normal human skin fibroblasts viability. The mechanical, morphology, UV-absorbance and thermal properties of the produced film have been evaluated.

## Materials and methods

### Materials

Bleached bagasse pulp was supplied from Quena Company of Paper Industry, Egypt, with chemical composition, cellulose (96%), hemicellulose (3%), and lignin (3%). Dulbecco's modified Eagle's medium (DMEM), [3-(4,5-dimethylthiazol-2-yl)-2,3-diphenyltetra zoliumbromide] (MTT), Sodium meta periodate (NaIO_4_), NaBr, and 2,2,6,6-tetramethylpiperidine-1-oxyl (TEMPO) were purchased from OXFORD LAB FINE CHEM LLP (Navghar, Vasai East, Maharashtra, India). Penicillin–streptomycin, amphotericin B, trypsin/EDTA solution, L-glutamine, and fetal bovine serum (FBS) were obtained from Invitrogen (Carlsbad, CA, USA). Doxorubicin HCl (Adricin^®^) was purchased from EBEWE Pharma (Unterach, AUSTRIA). Tissue culture flasks and tissue culture plates were purchased from Cole-Parmer(UK). All chemicals and reagents were used in analytical grade without any purification required before use.

### Methods

#### Synthesis of E-3-amino-5-(phenyldiazenyl)-2-thioxothiazolidin-4-one (TH)

In a reaction vessel, 5.1 mmol of sodium nitrite solution was dropped wisely for 10 min to the cooled solution of aniline (5 mmol) and 25 mL of aqueous hydrochloric acid solution (12 M, 32.19 mmol). The produced diazonium chloride was preserved at 0–5 °C; after that, an ethanolic solution (25 ml) of 2-thioxo-4-thiazolidinone (5 mmol) and sodium acetate (5 mmol) was added dropwise over 20 min. The resulting sludge mixture was stirred for 2 h at 5 °C before remaining standing overnight. The obtained dark brown precipitate (TH) was filtered, washed with water many times, and recrystallized by hot ethanol as shown in Fig. 2^28^, MS m/z: 252 (81%).

#### Oxidation of cellulose to dialdehyde cellulose (DAC)

Dialdehyde cellulose was prepared by oxidation of cellulose by sodium periodate under the effect of a microwave. Briefly, in 20 ml distilled water 1.5 g of cellulose was dispersed, followed by adding 2 g of sodium periodate, and pH was adjusted to 3. The precursor was transferred to the domestic microwave (power 800 W) for 1.5 min. The oxidized product (dialdehyde cellulose) was filtered, washed with ethanol several times, and dried^29^. The yield was calculated and it was 75% based on the weight of cellulose. Schiff's base reaction was used to calculate the aldehyde content of the oxidized cellulose, wherein aldehyde groups reacted with hydroxylamine hydrochloride forming an oxime. Va identifies the volume of alkali solution expended during the titration (in liter). The volume of 1.0 M sodium hydroxide solution consumed was presented as Vc, and the same concentration of cellulose solution at pH 5.0 was used as a blank (in liter)^5^:

$$Aldehyde\, Content \left(\%\right)=\frac{CNaOH\, X\, (Va-Vc)}{8\, X\, m/M}$$

In which CNaOH = 1.0 M, m denotes the dry mass of DAC (0.3 g) used during the experiment, and M denotes the molecular mass of the cellulose repeating unit (162).

#### Oxidation of cellulose to tricarboxylic cellulose (TCC)

Cellulose was oxidized to tricarboxy cellulose through three steps;

In the first step; to water-dispersed cellulose (5 g/ 500 mL), 0.08 g (0.5 mmol) of TEMPO reagent and 0.8 g (8 mmol) sodium bromide were added, followed by 50 mL (10%) of sodium hypochlorite solution and adjusting the pH to 10with stirring overnight. At the end of the reaction, the solution was neutralized and centrifuged at 7000 rpm, and washed with water. Finally, it was dialyzed for 1 week against water to complete the TEMPO-oxidized cellulose purification process. In the second step, sodium periodate was added to suspend TEMPO-oxidized cellulose (1% consistency). To prevent the photo-induced decomposition of the periodate, the reaction container was wrapped with aluminum foil. After 3 h, the reaction was terminated by adding ethylene glycol, washed with distilled water, and filtered, giving dialdehyde carboxy cellulose.

In the third step, a mixture of acetic acid (20%) and sodium chlorite solution was added slowly to a suspended dialdehydecarboxy cellulose (4.5% consistency), giving yellowish color and stirring for 48 h at ambienttemperature. Eventually, the induced tricarboxy cellulose (TCC) was In the third step, a mixture of acetic acid (20%) and sodium chlorite solution was slowly added to a suspended dialdehydecarboxy cellulose (4.5%), producing a yellowish colour and stirring for 48 h at room temperature. Eventually, the induced tricarboxy cellulose (TCC) was scrubbed with deionized water and filtered. TCC carboxylate content was calculated using the electric conductivity titration method by merging 50 mg of the dried sample with 0.01 M HCl (15 mL) and deionized water (20 ml). The mixture was stirred to achieve a well-dispersed suspension. Titration with 0.01 M NaOH aqueous solution was performed on the dispersed solution. The drastic shift in conductivity deduced the carboxylate content of TCC (C (mmol/g)) using the following Eq.:

$$C= \frac{\left(V1-V0\right)\,X\, CNaOH}{m}$$

where V1and V0 represent the volumes of NaOH solution before and after titration, CNaOH is the concentration of NaOH solution, and m is the weight of the dried sample^30,31^.

#### Reaction of DAC with TH

A suspended solution of DAC (1 g/ 20 ml distilled water) was added to a solution of TH (1 mol/ 30 ml of ethanol) and refluxed for 2 h with stirring. The resulted orange precipitate (DAC-TH) was filtered, washed several times with diluted ethanol, and dried at 80ºC for 24 h (yield ~ 60%).

#### Preparation of TCC/DAC-TH film

Different ratios of DAC-TH (2.5, 5, 7, and 10% based on the total weight of the film) were added into an aqueous dispersion TCC solution with drops of the glycerol as a plasticizer followed by sonication for 10 min, 30% P. The resulting TCC/DAC-TH suspension was poured into Teflon dishes and dried at 60 °C for 24 h.

### Characterization

FTIR spectra were performed on a Beckman infrared spectrophotometer PU 7712 using a KBr disk (United States). The scans recorded were the average of 100 scans and the selected spectral range between 500 and 4000 cm^−1^. The ^1^HNMR spectra were measured on Bruker AVANCE 400 MHz spectrometer (Bruker) with a 5 mm BBFO probe using deuterated dimethyl sulfoxide (DMSO-*d6*) as the solvent (Germany). Morphological studies have been measured using a scanning electron microscope (SEM) (Hitachi High Technologies America, Schaumburg, IL). The thermal behavior (TGA/DSC) is performed via (Perkin Elmer thermogravimetric analyzer (Waltham, Massachusetts, USA) under nitrogen conditions heating rate was 10 °C/min from 50 to 500 °C.

The mechanical properties of the prepared films were investigated using Universal Testing Machine model 4201 from Instron^33^. The stress–strain curve of films was tested on 6 cm film bars (width 15 mm; length 20 mm) with a Lloyd instrument (Lloyd Instruments, West Sussex, United Kingdom) with5-N load cell measurements at ambient temperature.

The UV-blocking properties of the TCC/DAC-TH films were performed by measuring their absorbance and total transmittance with a Shimadzu UV-3600 spectrometer from 200 to 2500 nm (UV–Vis–NIR spectrum, Japan). The T (UVA)and T (UVB) of the TCC/DAC-TH films were calculated using the following equations^14^:

$$T(UVA)\% = 100 - \frac{{\int_{320}^{400} {T_{\lambda } } \times d\lambda }}{{\int_{320}^{400} {d\lambda } }}$$

$$T(UVB)\% = 100 - \frac{{\int_{280}^{320} {T_{\lambda } } \times d\lambda }}{{\int_{280}^{320} {d\lambda } }}$$

The UV-protection factors (UPF) of films were measured by the AATCC 183–2010 using a UV–vis spectrophotometer, where T_λ_ is the light transmittance of TCC/DAC-TH films. The air was used as the control reference. UPF was measured from the transmission spectra of the films in the range of 290–400 nm using the following equation^34^:

$$UPF = \frac{{\sum\nolimits_{290}^{400} {E(\lambda ) \cdot S(\lambda )} }}{{\sum\nolimits_{290}^{400} {E(\lambda ) \cdot S(\lambda )} \cdot T(\lambda ) \cdot \Delta (\lambda )}}$$

where E(λ) is the relative erythemal spectral effectiveness, S(λ) is the solar spectral irradiance in Wm^−2^ nm^−1^. The values were provided by the National Oceanic and Atmospheric Administration database (NOAA)), T(λ) is the spectral transfer of the data taken from UV spectrophotometric tests, and Δ(λ) is the variation among observable wavelengths.

### Evaluation of the antibacterial activities

Using nutrient broth medium, the microbial activity of TCC/DAC-TH films was quantified against I Gram-negative bacteria: Escherichia coli (NCTC-10416); (ii) Gram-positive bacteria: Staphylococcus aureus (NCTC-7447); and (iii) unicellular fungi: Candida albicans (NCCLS 11). according to Hamed et al*.*^35^.The inhibition percentages were calculated for the crude antimicrobial material using the serial dilution method via colony formation unite (CFU) technique according to the procedure described by Abdelraof et al.^36^.

### Cell culture and determination of cell viability

Human skin fibroblastsHFB-4 cell line was obtained from the Egyptian National Cancer Institute, Cairo University, Egypt. The cells were maintained in DMEM media supplemented with 10% heat-inactivated FBS, 2 mMl L-glutamine, 100 IU/mL penicillin, 100 μg/mL streptomycin, and 0.25 μg/mL amphotericin B. Cultures were grown in 75cm2 tissue culture flasks at 37 °C in a humidified incubator with 5% CO2 and subcultured every 3–5 days.

Cells were treated with increasing concentrations of each treatment. As previously described, viability was calculated by monitoring the ability of reducing enzymes existing in viable cells to convert MTT to formazan crystals^37^. Briefly, HFB-4 cells were suspended in DMEM media supplemented with 10% heat-inactivated FBS and were plated in 96-well flat-bottom cell culture plates with a density of 3 × 10^4^ cell per well. The plates were incubated for 24 h at 37 °C in a 5% CO_2_ humidified incubator till 70% confluency. After that, media were replaced with serum free media containing 12.5, 25, 50, and 100 mcg/mL of each film, TCC, DAC-TH or doxorubicin as a positive control (100 µg/mL) that showed more 75% cytotoxicity, and the plates were incubated at 37 °C in a CO_2_ humidified incubator. Twenty-four h later, media were removed, and 100 μL of MTT solution (1.2 mM in PBS) was added to each well. After 4 h incubation, isopropyl alcohol (100 μL) was added to each well, and the plates were placed in a plate shaker for 1 h. Using a BIO-TEK Instruments EL 312e microplate reader (Bio-Tek Instruments, Winooski, VT), the blue color constituted in each well was estimated at 570 nm with a reference wavelength of 690 nm. Results were expressed as a percentage of cell viability relative to DMSO-treated control wells designated as 100% viable cells, and the experiment was repeated 2 times.

### Statistical analysis

Cell viability data are summarized as mean ± S.E.M., and statistical analysis for viability bar graphs per each experimental group was performed using one-way ANOVA pursued by Student–Newman–Keuls post hoc test in Sigma Stat 3.5 for Windows, Systat Software Inc. (San Jose, CA).

### Ethical approval

The cell experiments were consistent with the protocols adopted by the local ethical committee (Ethical Committee of National Research Centre).

## Results and discussion

### Chemistry

**Figure 1:**
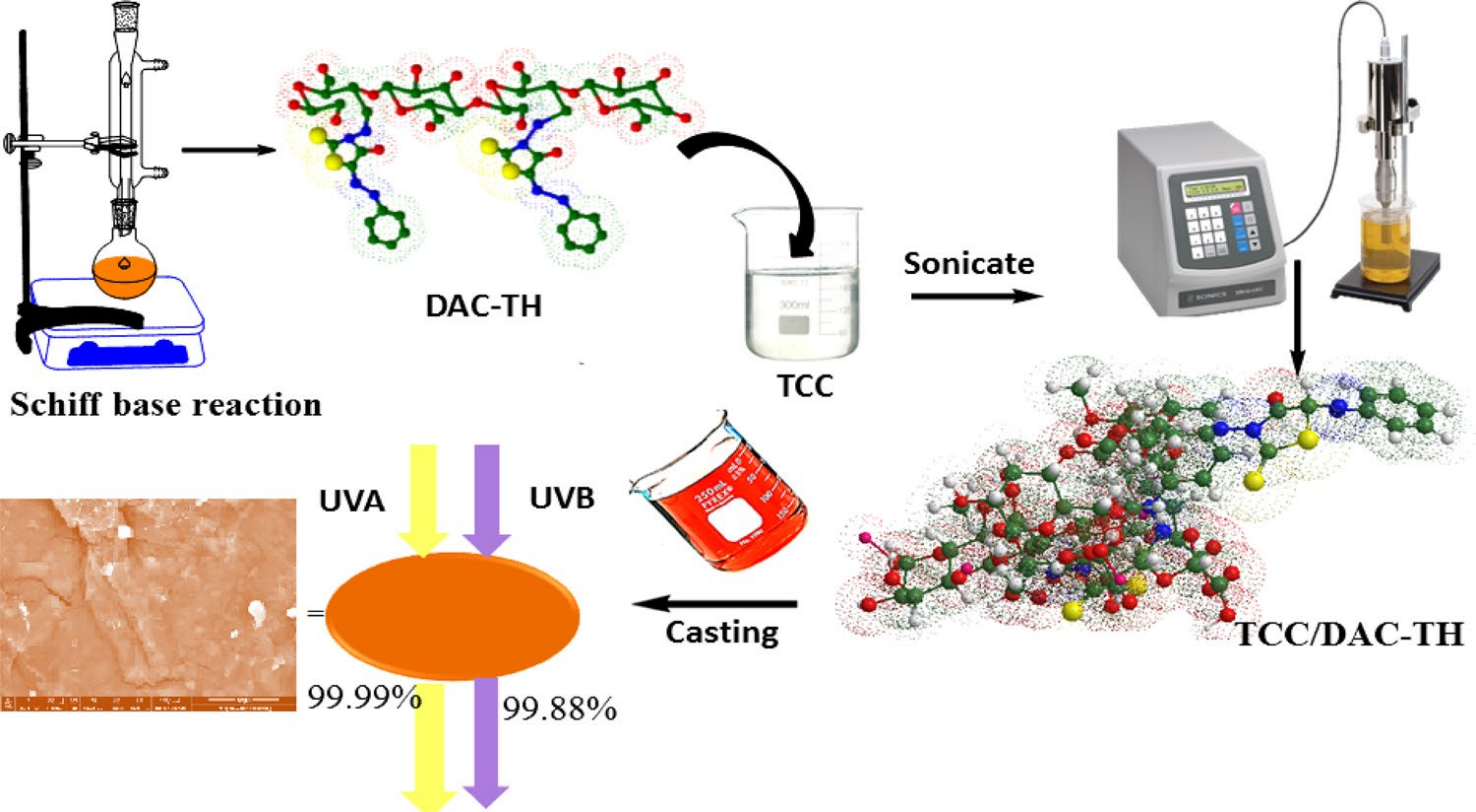
Graphical abstract.

**Figure 2:**
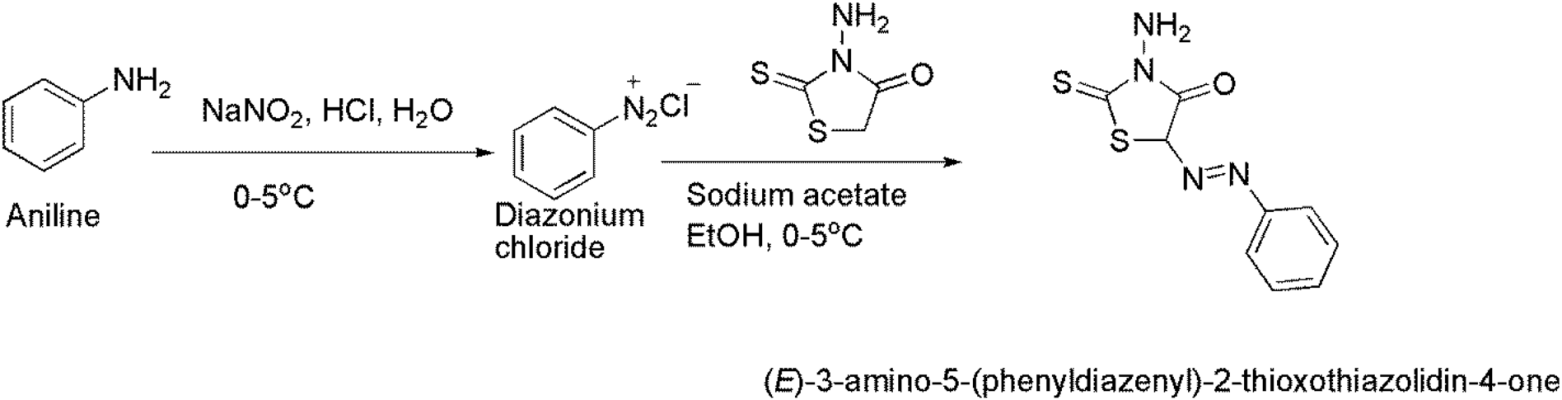
Synthetic route of TH.

**Figure 3:**
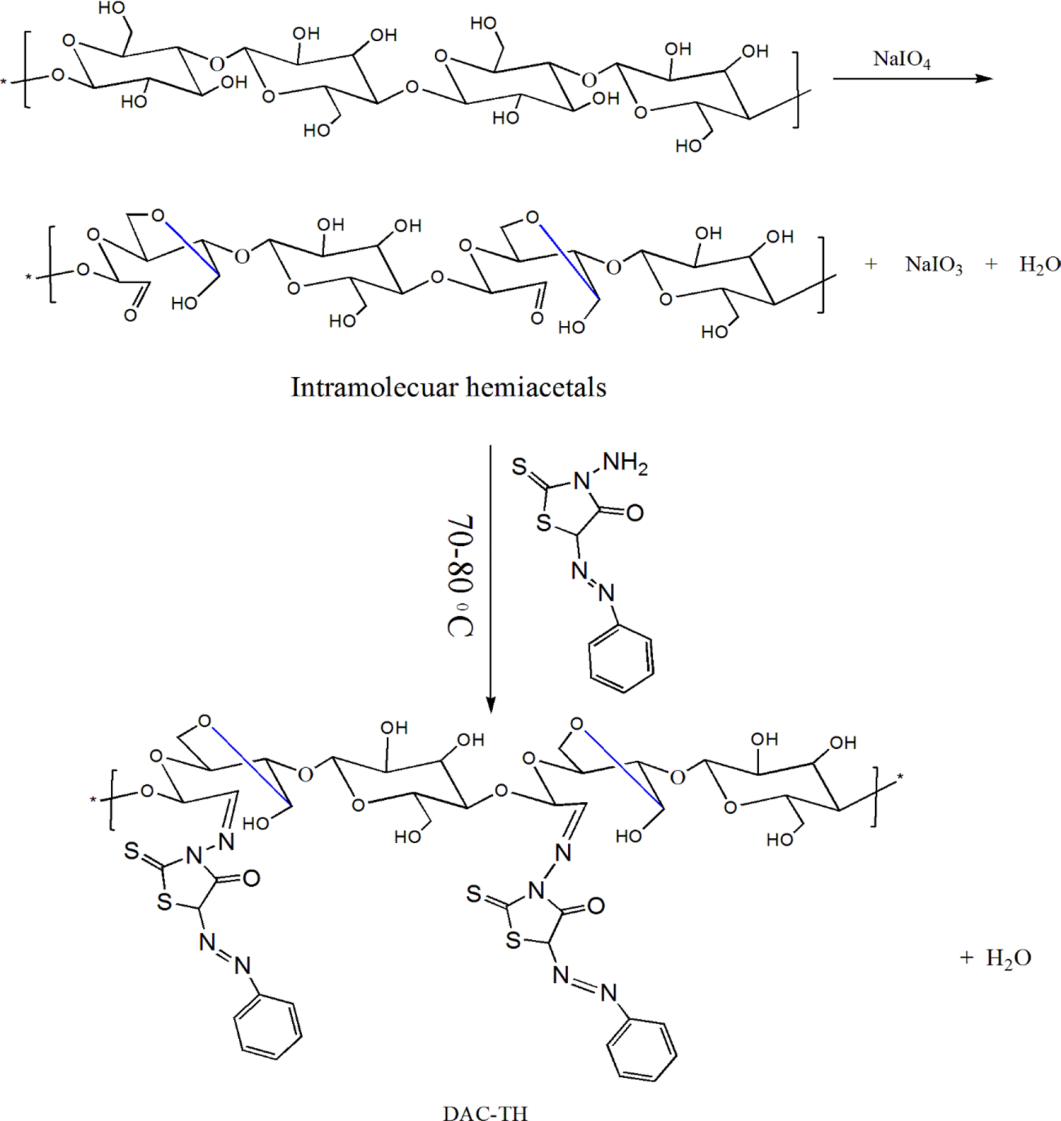
Plausible mechanism of oxidation of cellulose to DAC, the reaction of DAC with TH^32^.

**Figure 4:**
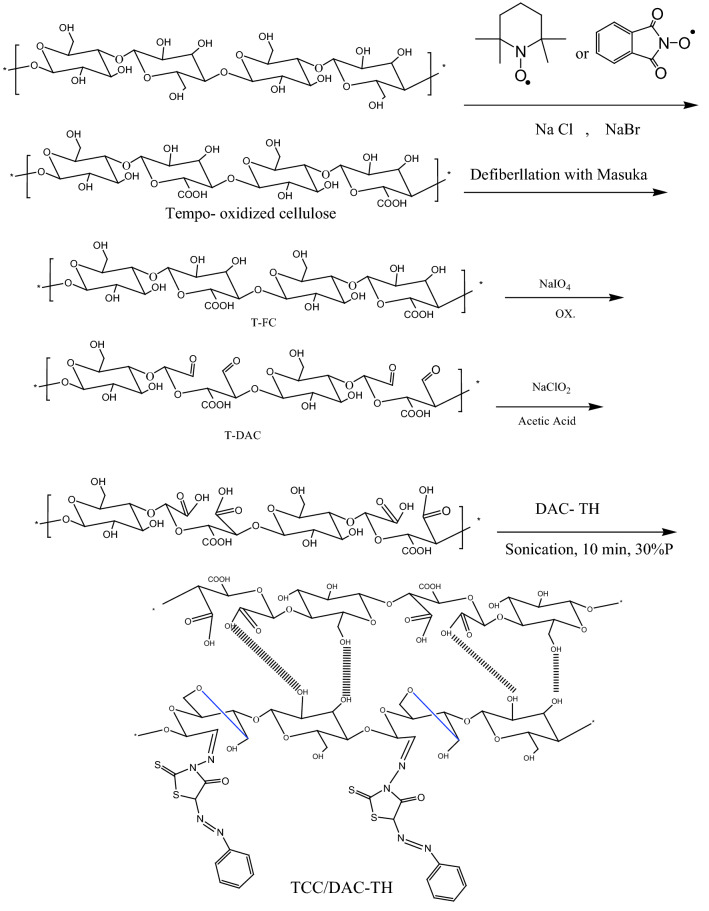
Plausible mechanism of oxidation of cellulose to TCC, and formation of TCC/DAC-TH film.

As presented in Fig. 1 TCC/DAC-TH film was prepared in several steps and evaluated as a UV-shielding, where the assumed synthetic mechanisms of TH, DAC, DAC-TH, and TCC/DAC-TH films are outlined in Figs. 2, 3, 4. TH was synthesized from the reaction of the diazonium salt with the 5-methylene of 2-thioxo-4-thiazolidinone through a coupling reaction (Fig. 2) with a yield of 81%. As apparent in Fig. 3, DAC has successfully synthesized via periodat oxidation with dialdehyde content 60%. As is well known, reactive aldehyde functionalities can exist as hydrates, hemialdals, and hemiacetals^32^. The intra-chain and intra-anhydroglucose unit hemiacetal structure are feasible among C2/C6 and C3/C6 and hemialdal C2/C3^38^. In addition, DAC-TH was prepared via a Schiff base reaction as presented in Fig. 3. An amine reacted with a carbonyl compound (DAC) via nucleophilic addition, and then dehydration formed hydrated cellulose aldehyde. Also, according to a previous study, Scheme 3 defines the suggested mechanism of formation of TCC with carboxyl contents of 2 ± 0.3 mmol/g^30^. In the presence of sodium bromide and sodium hypochlorite, TEMPO performs as a mediator as selectively oxidation of the primary hydroxyl groups (C6). After that, hydroxyl groups in C2 and C3 were oxidized to the aldehyde groups using periodate, which further oxidized to generate TCC.

**Figure 5:**
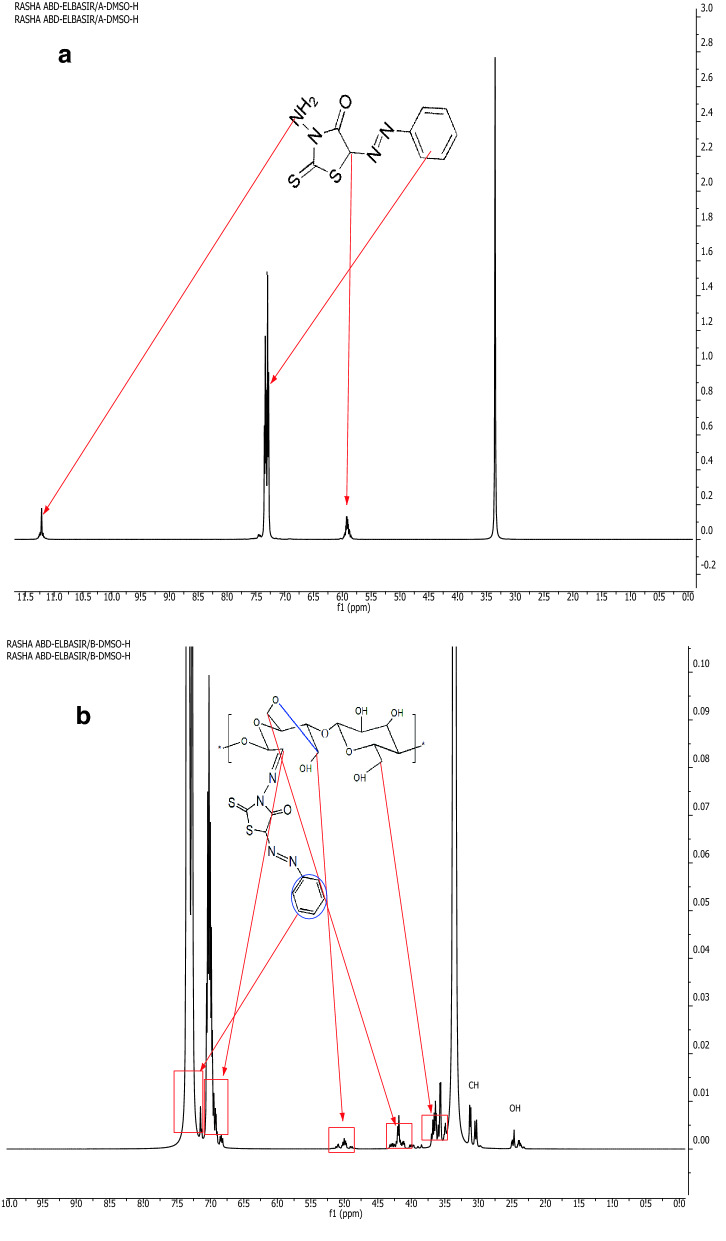

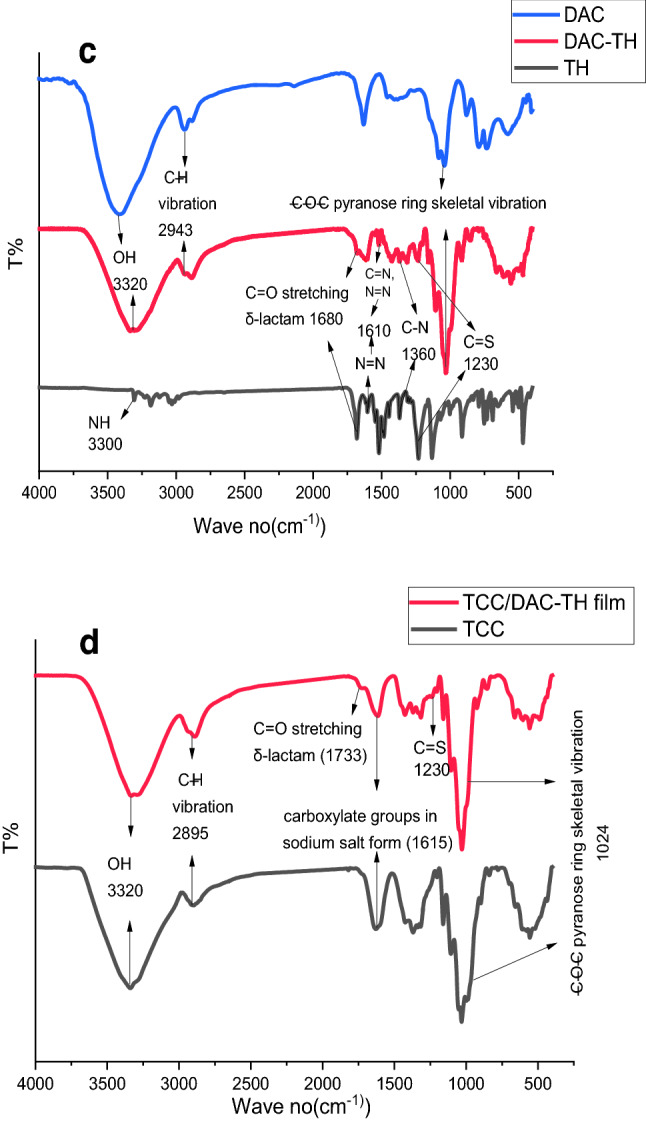
^1^HNMR of; (**a**) TH and (**b**) DAC-TH. FTIR Spectra of; (**c**) DA, TH, DA-TH, TCC, and (**d**) TCC/DAC-TH films.

The afforded TH, DAC, and DAC-TH were confirmed by ^1^HNMR and FT-IR (Fig. 5). As evident, ^1^HNMR of TH reveals the presence of peaks at δ 11.22 (s, 2H, NH_2_), 7–7.36(m, 5H, Ar–H), and 5.92(s, 1H, CH thiazole). On the other hand,^1^HNMR of DAC-TH induces the presence of peaks at δ 7.28(m, 5H, Ar–H),6.99 (m, CH, hydrazide), 4.99 (m, 1alpha –O from methine), 4.20(m, 1beta –O–C from methylene), 3.58(m, CH_2_), 3.02(m, CH glucose), and 2.38(m, 1H, OH). Also, Fig. 5 shows significant FTIR spectra for TH, DAC, DAC-TH, TCC, and TCC/DAC-TH film. FTIR spectra of DAC, TH, and DAC-TH reveal a band at 3320 cm^−1^ related to (OH) for both DAC and DAC-TH^39^, a band at 3300 cm^−1^ assigned to (NH_2_) of TH^40^, a band at 2943 cm^−1^ corresponding to (C–H vibration), 1680 cm^−1^ regarding C=O stretching δ-lactam, the band at 1630 cm^−1^ is due to C=O in DAC, due to hemiacetal bond formation (between aldehyde groups and their adjacent hydroxyl groups OH), so sometimes this peak can be very small and hidden due to presence DAC in the hydrated form^41,42^. The band at 1610 cm^−1^ regarding to both (N=N) and (C=N)^43^, 1360 cm^−1^ for C–N, 1230 cm^−1^ for C=S^43^and 1030 cm^−1^ related to –C–O–C pyranose ring skeletal vibration^44^. Also, bands at 1733, 1615, 1230, and 1024 cm^−1^ attributed to stretching δ-lactam, carboxylate groups, C=S, and –C–O–C pyranose ring skeletal vibration, respectively^40,44^ (Fig. 5).

### Thermogravimetric analysis

**Figure 6:**
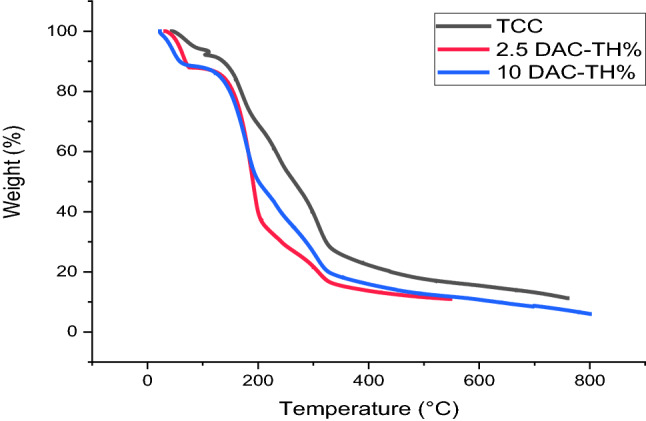
TGA of TCC films with 0, 2.5, and 10% DAC-TH.

The thermogravimetric analysis (TGA) of TCC films with 0, 2.5, and 10% DAC-TH is presented in Fig. 6. As apparent, three stages of degradation are observed for both TCC and TCC/10%DAC-TH. At the first stage of TCC thermal degradation, the wt loss is about 7% at 68–169 °C, attributed to water evaporation. The second stage is the degradation initiation temperature of TCC, which occurs from 169–330 °C and the wt loss is 47% due to the formation of CO_2_, H_2_O, and CO^35^. Finally, the residual solid is approximately 35% at the final stage ranging from 330–600 °C. In comparison, TCC/10%DAC-TH shows 11% wt loss in the region from 47–179 °C for water evaporation and 53% wt loss at 179–308 °C due to DAC oxime^45^. Finally, the residual solid is approximately 35% at the final stage ranging from 330°–600 °C. At the same time, TCC/10%DAC-TH shows 11% wt loss in the region from 47°–179 °C for water evaporation and 53% wt loss at 179–308 °C due to DAC oxime^46^ and azo degradation besides the formation of CO_2_, H_2_O, and CO. The final residual solid is approximately 30% in the region from 308–600 °C. Eventually, TCC/2.5%DAC-TH shows four degradation stages despite TCC/10% DAC-TH. That may explain by increasing the crosslinking results from hydrogen bonding between TCC and DAC as DAC concentration increases. Accordingly, TCC/DAC-TH shows high thermal stability at a higher concentration of DAC-TH rather than less concentration. In comparison, TCC/2.5% DAC-TH shows 12% wt loss for water evaporation at a temperature from 64–190 °C and 26% wt loss at 190–232 °C due to DAC oxime azo degradation, 36% wt loss at 232,311 °C to the formation of CO_2_, H_2_O, and CO. The final TCC/2.5% DAC-TH residual solid is about 15% at 311–600 °C.

### Morphology and energy-dispersive X-ray spectroscopy (SEM/EDX) analysis

**Figure 7:**
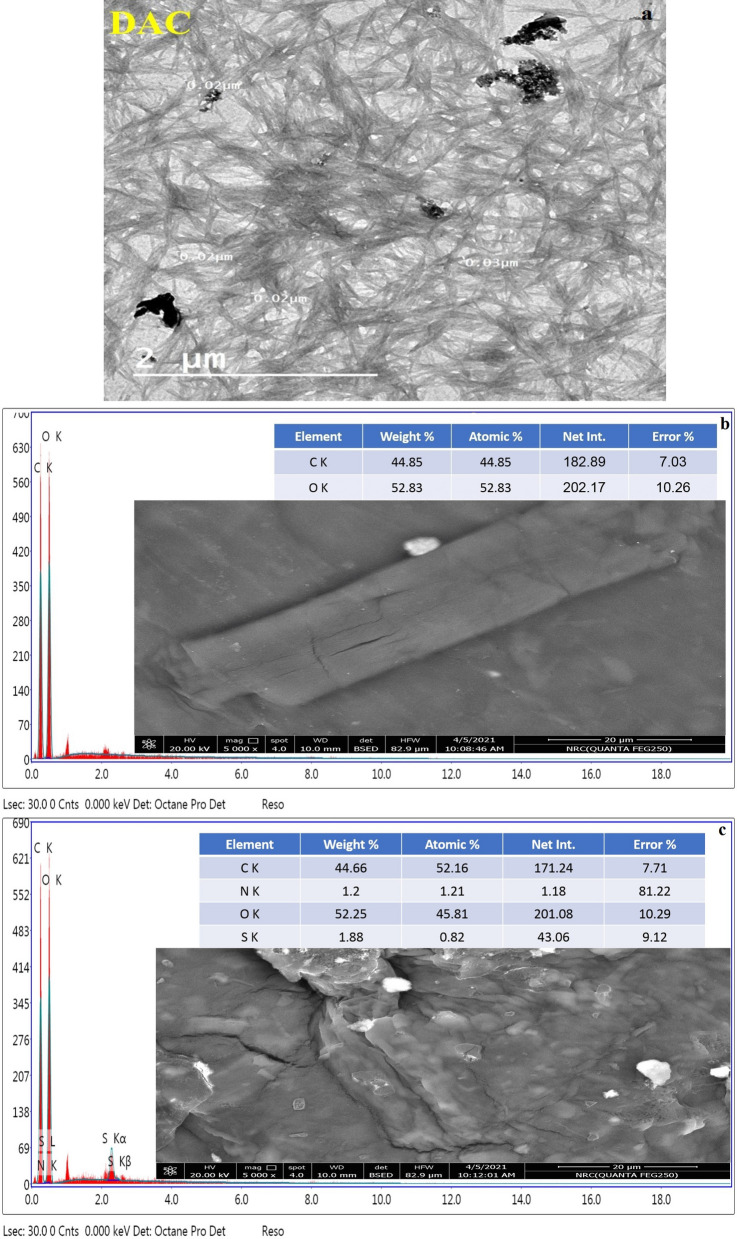
SEM images with EDX analysis of (**a**) DAC fiber, (**b**) TCC, and (**c**) TCC/DAC-TH films.

The morphological structure of DAC fiber, TCC, and TCC/DAC-TH films are characterized via scanning electron spectroscopy (SEM). As presented in Fig. 7, DAC fiber seems like a hair cluster structure, while TCC film showed a smooth surface. Furthermore, TCC/DAC-TH film showed a rough, thick layer of DAC-TH with semi-uniform dispersion. On the other side, the existence of DAC-TH was confirmed by Energy Dispersive X-ray spectroscopic analysis (EDX). EDX ensures the significant elements of DAC-TH, where the EDX analysis of TCC/showed two signals related to N and S opposite what TCC/DAC-TH film presents.

### Mechanical properties

**Figure 8:**
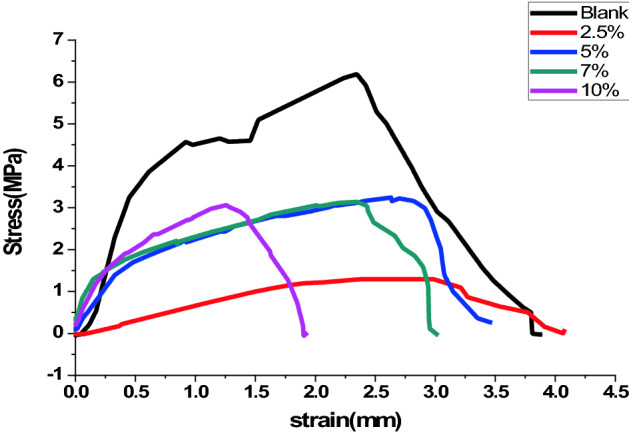
Stress/ strain curves of TCC/DAC-TH films with different ratios of DAC-TH.

The ability of films to withstand external stress is one of the essential features of films, and mechanical properties control this ability Fig. 8 shows the stress/strain curves of TCC/DAC-TH films with different ratios of DAC-TH that were investigated at room temperature. As it seems, increasing DAC-TH ratios decreases the tensile strength and elongation of the prepared films. For example, tensile strength and elongation of TCC film were 6.5 MPa and 4 mm and decreased to 2.5 MPa and 2 mm, respectively, with the addition of DAC-TH. It may be attributed to crosslinking formation between TCC and DAC-TH that causes structure deformation; as a result, the crystallinity of the films decreases.

### UV-shielding performance

**Figure 9:**
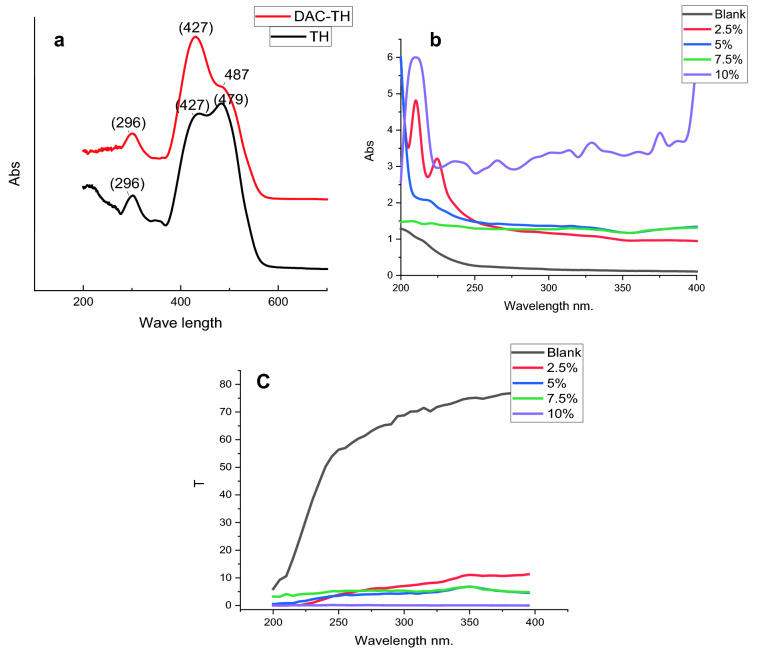
UV absorbance of (**a**) TH and DAC-TH in DMSO TCC, (**b**) and TCC/DAC-TH films, and (**c**) transmittance of TCC/DAC-TH films with different ratios of DAC-TH.

UV absorbers have been widely used for UV-shielding improvement of films as the indispensable demand in several packaging industries^47–49^. As previously known, heterocyclic compounds have significant UV absorption properties^20,34,50^. Therefore, TH has been used to graft DAC and incorporated the resulted DAC-TH into TCC with different ratios to evaluate their UV-shielding features. Figure 9a presents the UV absorbance of TH and DAC-TH in DMSO using a Shimadzu UV–vis-2401 PC from 200–700 nm. It is known that the absorption spectrum of azobenzene and its derivatives shows three major absorption regions accordingly to the basis of Molecular Orbital Theory (MO)^51^. Where the solution of TH and DAC-TH showed main absorption peaks at 296and 427 nm for both of them due to π → π^*^ transition and locally excited π_i_ → π_j_^*^ transition in benzene rings. Moreover, the two nitrogen atoms of the azo group exhibit two lone-pair orbitals that overlap, arising from the splitting of the originally degenerate n orbitals where the higher and lower energy orbitals are symmetric combination (ns) antisymmetric combination (n_a_), respectively. Eventually, TH and DAC-TH solutions exhibit absorption bands at 479and 487 nm, respectively, which may be attributed to the n_s→_π_i_ transition^52^, where it seems weak for DAC-TH, maybe back to the overlap results from the aldehyde oxime of DAC.

The films' UV absorbance and transmittance curves are shown in Fig. 9b,c, which clarify the impact of increasing the DAC-TH ratio on the UV shielding properties. According to the equations, the transmittance of UV-A (320–400) and UV-B (280–320) were implemented to explore UV Shielding properties. The obtained results indicated that TCC/DAC-TH films showed an excellent UV shielding capacity at which the film with the highest DAC-TH ratio (10%) has the highest UV transmittance of UVA (99.99%) and UVB (99.88%). Therefore, DAC-TH has excellent sensitivity toward UV light and can absorb light ranging from 200 to 400 nm. On the other side, the film's transparency descends with increasing the DAC-TH ratio.

**Figure 10:**
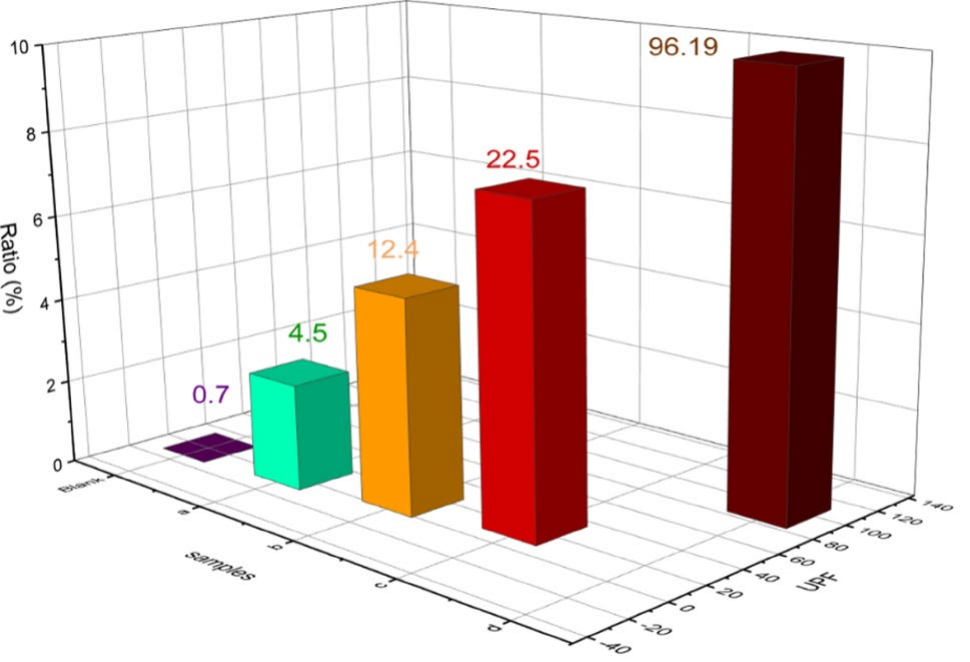
UV protection of TCC/DAC-TH films with different ratios of DAC-TH.

On the other hand, the UV-protection (UPF) study of TCC/DAC-TH films agrees with the UV-shielding study. As clear in Fig. 10, DAC-TH addition shows UV protection compared without DAC-TH. Herein, TCC/DAC-TH film of 10% DAC-TH has excellent UV protection according to standard methods. The evolution of UPF values between 0–50 while between 15–24 is good, 25–39 is very good, and > 40 is excellent UV protection.

### Evaluation of the antibacterial activities

The antimicrobial activity screening of thiazolidine derivatives against common bacteria and fungi revealed a high inhibition effect^53,54^. Consequently, the thiazolidine derivative such as TH was coupled with DAC giving DAC-TH, and to formulate this derivative, it was loaded onto TCC to give films. The efficiency of antimicrobial activity of TCC/DAC-TH films with different ratios of DAC-TH on *Escherichia coli, S. aureus,* and *Candida albicans* has been distinguished by the CFU technique as described previously (Table 1). In this respect, TCC film without DAC-TH did not show any antimicrobial effect. In contrast, TCC films with DAC-TH had an antimicrobial effect for all tested microorganisms, which was due to TH being characterized by antimicrobial activity. Dandia et al*.* recommended that N–C–S linkage is responsible for the antifungal activity of thiazolidine derivatives^55^.

**Table 1:** Antimicrobial susceptibility of the TCC/DAC-TH films with different DAC-TH ratios by using the CFU method.

DAC-TH (%)	*Escherichia coli* (*NCTC-10416*)	*Staphylococcus aureus(NCTC-7447)*	*Candida albicans (NCCLS 11)*
0.0	0.0	0.0	0.0
2.5	9 ± 0.81	8 ± 0.76	12 ± 0.85
5.0	23 ± 1.09	16 ± 1.21	23 ± 1.29
7.5	31 ± 2.91	22 ± 1.78	61 ± 3.41
10.0	72 ± 3.71	64 ± 2.55	66 ± 3.70

Moreover, increasing DAC-TH content in TCC films inhibited the growth of bacteria and fungi. In contrast, with the same DAC-TH content in films, the films have a highly inhibited effect on *Escherichia coli* than *Staphylococcus aureus*. The rate of inhibition of fungi (Candida albicans) was higher than that of bacteria. The results showed that the 10% DAC-TH content inhibited 72, 64, and 66% of *Escherichia coli, Staphylococcus aureus, and Candida albicans*.

### Determination of cell viability

**Figure 11:**
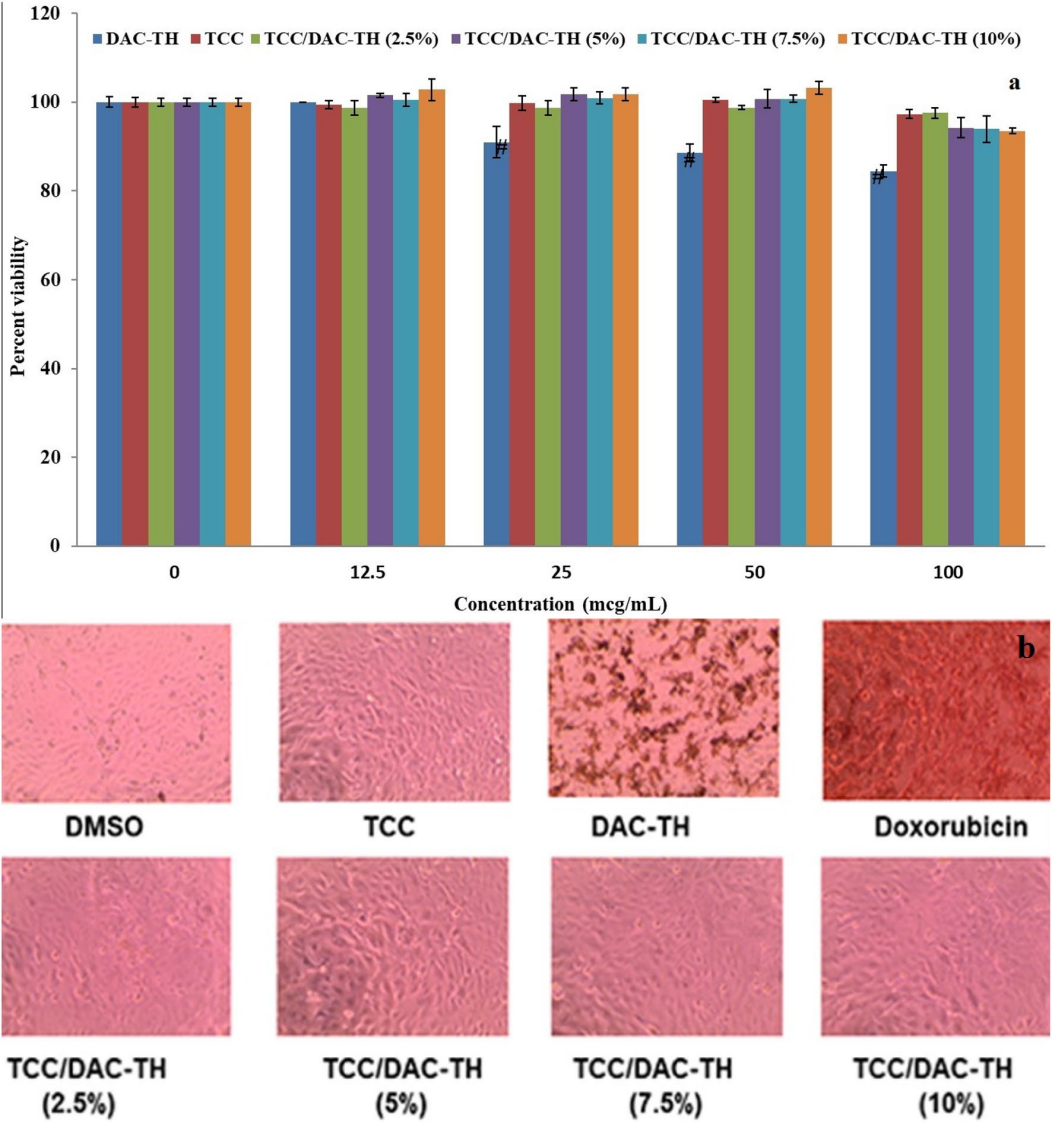
(**a**) Effect of prepared film formulations on HFB-4 cell viability using the MTT assay. (**b**) The photographs of HFB-4 cells with different treatments at 100 mcg/mL for 24 h, with a light inverted microscope 100 X.

To test whether the prepared films can affect cell viability, we chose human skin fibroblasts HFB-4 cells. HFB-4 cells have been used previously to study the harmful effect of UV radiation and to detect the role of UV protecting agents^56^. As indicated in Fig. 11, all prepared films and TCC showed no cytotoxicity up to 100 mcg/mL when incubated with HFB-4 cells for 24 h. In contrast, DAC-TH showed moderate cytotoxicity that was statistically significant compared to vehicle-treated cells starting from 25 mcg/mL (Fig. 7a). Photographs were captured for all treatments at 100 mcg/mL, and the positive control, doxorubicin, after 24 h incubation period that reflected the safety of all the prepared film formulations (Fig. 11b).

## Conclusion

In conclusion, a new antimicrobial/ UV protection film based on cellulose and *E*-3-amino-5-(phenyldiazenyl)-2-thioxothiazolidin-4-one was successively prepared. In addition to film preparation, the properties of the films, such as UV protection, mechanical, thermal, and biological, were studied. A high degree of UV protection appeared with 10% dialdehyde cellulose. The prepared films have demonstrated significant antibacterial activities and did not affect the cell viability of human skin fibroblasts. Thus, this product is synthetically important and possesses a wide range of promising applications like food packaging, skincare materials, and electronic device coating.

## Data Availability

All data generated or analyzed during this study are included in this article.
